# Supplementary material for: Rational combination of oncolytic vaccinia virus and PD-L1 blockade works synergistically to enhance therapeutic efficacy
Source: Nat Commun. 2017 Mar 27;8:14754. doi: 10.1038/ncomms14754 (PMC5378974; doi:10.1038/ncomms14754)
Supplement: Supplementary Information — Supplementary Figures [file ncomms14754-s1.pdf]

Supplementary Figures

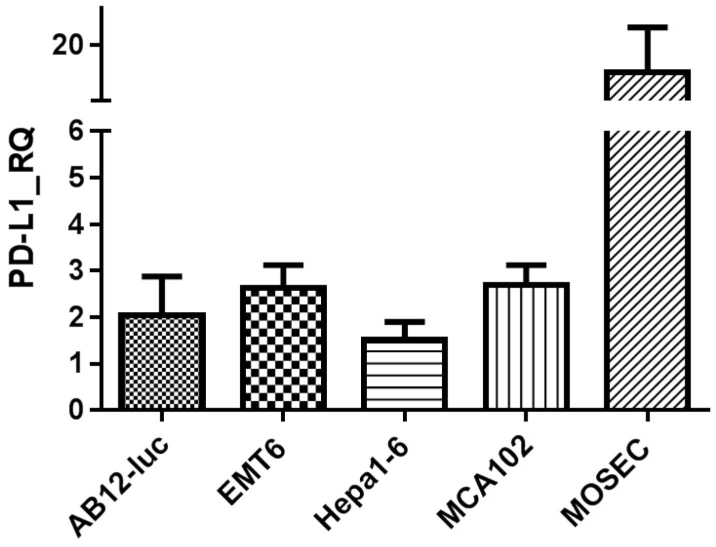

Supplementary Figure 1. PD-L1 is elevated post vvDD treatment in other murine cancer cells *in vitro*. Murine AB12-luc, EMT6, Hepa1-6, MCA102, and MOSEC cancer cells were infected with vvDD. The infected cells were harvested 24 h post infection to make RNA for RT-qPCR assay to determine relative PD-L1 expression.

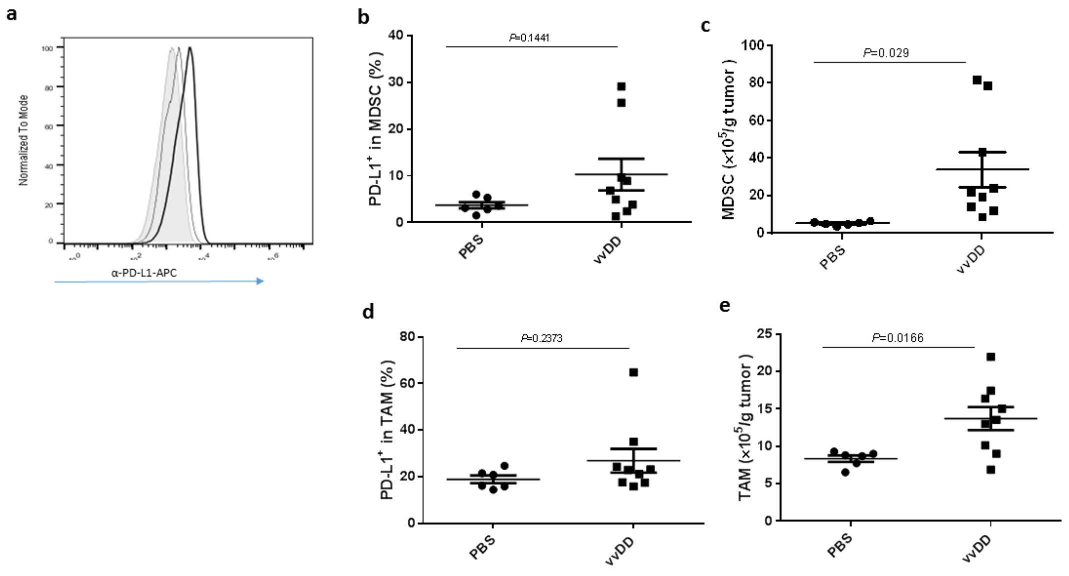

Supplementary Figure 2. PD-L1 is impacted in MDSC and TAM post vvDD treatment *in vivo*. B6 mice were subcutaneously inoculated with MC38-luc cells (4×10^5 cells). PBS or vvDD was intratumorally injected at 2×10^8 pfu/tumor when the s.c tumor area reached 5x5 mm^2. Tumor tissues were collected from PBS or virus-treated mice 4 days post the treatment. Collected tumor tissues were weighed and incubated in RPMI 1640 containing 2% FBS, 1 mg/ml collagenase, 0.1 mg hyaluronidase, and 200 U DNase I at 37°C for 1-2h to make single cells. The single cells were blocked with α-CD16/32 Ab and then stained with antibodies against CD45, CD11b, Gr1, PD-L1, F4/80 to determine PD-L1 expression on $CD45^-$ cells, tinted: Ab isotype; (a). hairline: PBS-treated; normal: vvDD-treated; (b,c). MDSC ($CD45^+CD11b^+Gr1^+$); (d, e); TAM ($CD45^+CD11b^+F4/80^+$). Student's *t*-test was used to analyze the data.

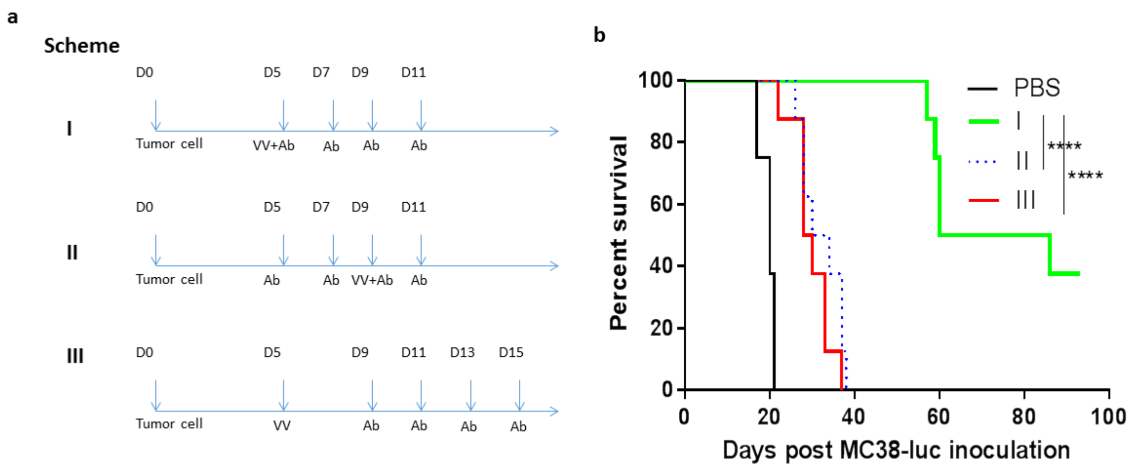

Supplementary Figure 3. Simultaneous treatment with VV-CXCL11 plus α-PD-L1 is more effective at early treatment time points. (a). B6 mice were intraperitoneally inoculated with 5×10^5 MC38-luc and treated with VV-CXCL11 + α-PD-L1 as per the timelines, and (b). The survival data was shown . The survival was monitored by Kaplan-Meier analysis and statistical analyses were performed with Log rank test. In all of the figures, the standard symbols for *P* values are, * *P*<0.05; ** *P*<0.01; *** *P*<0.001; and **** *P*<0.0001; and ns: not significant.

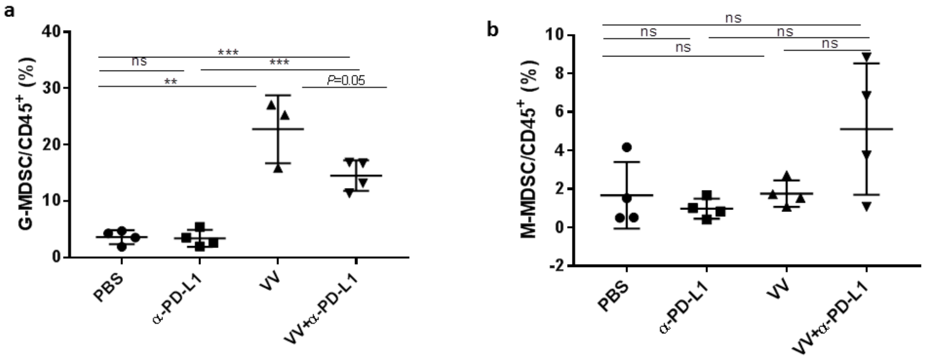

Supplementary Figure 4. Vaccinia virus treatment elevates G-MDSC in TME. B6 mice were intraperitoneally inoculated with 5×10^5 MC38-luc cells and treated with VV (short-named for vvDD-CXCL11) and/or α-PD-L1 as described. Tumor-bearing mice were sacrificed at day 5 post first treatment and primary tumors were collected and analyzed as Figure 5 for G-MDSC and M-MDSC in TME. Student's *t*-test was used to analyze the significance of the differences.

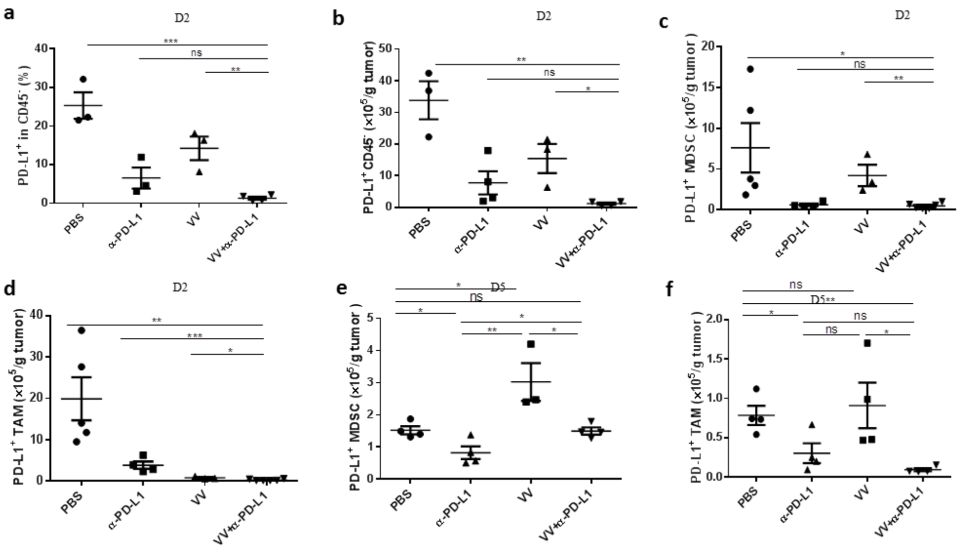

Supplementary Figure 5. The α-PD-L1 treatment reduces the PD-L1^{+} cells in TME. C57BL/6 mice were intraperitoneally inoculated with 5×10^{5} MC38-luc and treated with VV-CXCL11 and/or α-PD-L1 as described. Tumor-bearing mice were sacrificed at day 2 or day 5 post first treatment and primary tumors were collected and analyzed as Figure 2 to determine the PD-L1^{+} tumor cells (a,b), PD-L1^{+} MDSC (c, e) PD-L1^{+} TAM (d, f) in the TME. Student's *t*-test was used to analyze the significance of the differences.

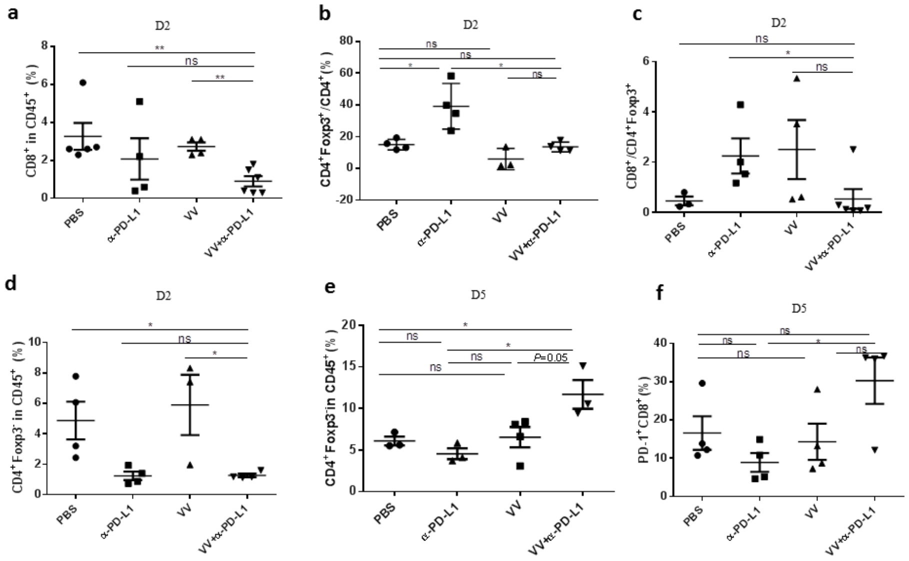

Supplementary Figure 6. The combination of VV plus α-PD-L1 treatment impacts tumor-infiltrating effector T cells in TME. B6 mice were intraperitoneally inoculated with 5×10^5 MC38-luc and treated with VV and/or α-PD-L1 as described. Single cells were made from primary tumors collected from tumor-bearing mice at day 2 or day 5 post first treatment as Figure 2, blocked with α-CD16/32 Ab and then stained with antibodies against CD45, CD8, CD4 and Foxp3 to determine the $CD8^+$ T cells (a), $CD4^+Foxp3^+$ (b), $CD8^+/CD4^+Foxp3^+$ (c), $CD4^+Foxp3^-$ (d, e) and PD-1^+ $CD8^+$ (f) in the TME. Student's *t*-test was used to analyze the significance of the differences.

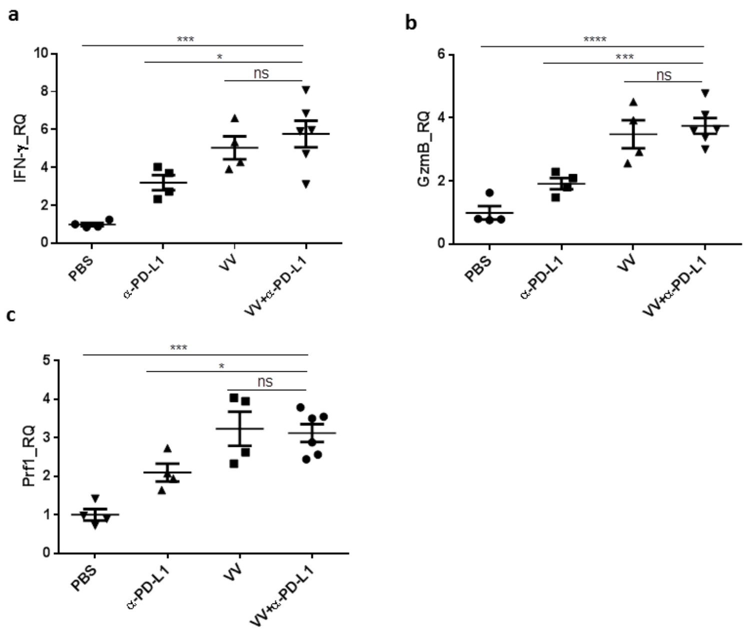

Supplementary Figure 7. The combination therapy of VV plus α-PD-L1 increases immune-favorable molecules in the TME. B6 mice were intraperitoneally inoculated with 5×10^5 MC38-luc and treated with VV-CXCL11 and/or α-PD-L1 as described. RNA extracted from tumor tissues collected from tumor-bearing mice at day 2 post first treatment was used for RT-qPCR assay to determine IFN-γ (a), Granzyme B (b) and perforin (c) in the TME. Student's *t*-test was used to analyze the significance of the differences. The standard symbols for *P* values are, * $P<0.05$; ** $P<0.01$; *** $P<0.001$; and **** $P<0.0001$.
